# Attention to Eye Contact in the West and East: Autonomic Responses and Evaluative Ratings

**DOI:** 10.1371/journal.pone.0059312

**Published:** 2013-03-13

**Authors:** Hironori Akechi, Atsushi Senju, Helen Uibo, Yukiko Kikuchi, Toshikazu Hasegawa, Jari K. Hietanen

**Affiliations:** 1 Department of Cognitive and Behavioral Science, University of Tokyo, Tokyo, Japan; 2 Centre for Brain and Cognitive Development, Birkbeck, University of London, London, United Kingdom; 3 Institute of Psychology, University of Tartu, Tartu, Estonia; 4 Human Information Processing Laboratory, School of Social Sciences and Humanities, University of Tampere, Tampere, Finland; Durham University, United Kingdom

## Abstract

Eye contact has a fundamental role in human social interaction. The special appearance of the human eye (i.e., white sclera contrasted with a coloured iris) implies the importance of detecting another person's face through eye contact. Empirical studies have demonstrated that faces making eye contact are detected quickly and processed preferentially (i.e., the eye contact effect). Such sensitivity to eye contact seems to be innate and universal among humans; however, several studies suggest that cultural norms affect eye contact behaviours. For example, Japanese individuals exhibit less eye contact than do individuals from Western European or North American cultures. However, how culture modulates eye contact behaviour is unclear. The present study investigated cultural differences in autonomic correlates of attentional orienting (i.e., heart rate) and looking time. Additionally, we examined evaluative ratings of eye contact with another real person, displaying an emotionally neutral expression, between participants from Western European (Finnish) and East Asian (Japanese) cultures. Our results showed that eye contact elicited stronger heart rate deceleration responses (i.e., attentional orienting), shorter looking times, and higher ratings of subjective feelings of arousal as compared to averted gaze in both cultures. Instead, cultural differences in the eye contact effect were observed in various evaluative responses regarding the stimulus faces (e.g., facial emotion, approachability etc.). The rating results suggest that individuals from an East Asian culture perceive another's face as being angrier, unapproachable, and unpleasant when making eye contact as compared to individuals from a Western European culture. The rating results also revealed that gaze direction (direct vs. averted) could influence perceptions about another person's facial affect and disposition. These results suggest that cultural differences in eye contact behaviour emerge from differential display rules and cultural norms, as opposed to culture affecting eye contact behaviour directly at the physiological level.

## Introduction

The importance of eyes for daily social communication is portrayed by several ‘eye’ metaphors across cultures (e.g., ‘Eyes are the windows to the soul’ in the West and ‘Eyes are as eloquent as the tongue’ in Japan). Human eyes have a wide, depigmented (i.e., white) sclera that is contrasted with a coloured iris, a characteristic which does not exist in other primate species. This contrast makes it easy to detect another person's gaze direction. It has been suggested that the special appearance of the human eye is an adaptive consequence that implies the importance of detecting eye contact for social interaction [1]. Both faces displaying direct and averted gaze have crucial functions for social interaction. Faces displaying a direct gaze capture our visual attention, whereas faces displaying an averted gaze trigger shifts in attention toward the averted location (for a review, see [2]). The importance of eye contact is also supported by research indicating that information detected from another person's eye gaze is processed in specific brain areas, such as the amygdala and superior temporal sulcus (STS; for reviews, see [3], [4]). Moreover, from a very early age, human children are sensitive to others' eye contact, suggesting that there is an innate sensitivity to others' eye gaze. For example, even newborns (2–5 days old) look at faces displaying a direct gaze longer than faces displaying an averted gaze [5], [6]. Faces displaying direct eye contact are treated specially in adults, as well. For example, faces displaying a direct gaze are detected faster [7], [8], these faces hold our visual attention [9], and these faces increase an observer's autonomic arousal (e.g., skin conductance response, [10]; heart rate, [11]) more than faces displaying an averted gaze. Generally, humans tend to rate a person who makes eye contact as more likable, pleasant, intelligent, credible, and dominant as compared to a person exhibiting less or no eye contact. However, excessive eye contact may make an observer feel uncomfortable in certain situations ([12] for a review). Faces displaying a direct gaze enhance performance on face-related tasks, such as gender discrimination [13] and encoding and decoding of facial identity [14].

Although sensitivity to socially significant stimuli, such as faces and eye contact, is innate to humans, there is also some variability across cultures. For example, it has been reported that culture affects the speed of facial age judgements; Japanese participants judge the age of East Asian children's faces faster than do Chinese and Asian-Canadian participants [15]. It has also been shown that culture affects holistic face processing; White people process own-race faces more holistically than East Asian faces, whereas East Asians holistically process both own- and other-race faces [16]. Some researchers argue that facial recognition and expression of basic emotions is universal among humans (e.g., [17], [18]); however, there is also evidence that culture influences how individuals process others' faces (e.g., [19]). Specifically, East Asians tend to categorise fearful faces as surprised faces [20]–[22] and disgusted faces as angry faces [20]. A recent study demonstrated that cultural differences in recognition of facial expressions of fear and disgust might be due to differences in eye fixation patterns [20]; Western Europeans fixate more on the mouth region, and East Asians fixate more on the eye region when recognising facial expressions. Another study reported that Japanese people tend to use information from the eyes, and U.S. people rely on the mouth when recognising emotion in faces [23]. Even within Western European and North American cultures, there is variability in the recognition of facial expression (e.g., [24]). A meta-analysis showed that there is an in-group advantage in recognising facial expressions (i.e., emotional facial expressions of individuals in the same race/culture are easier to recognise as compared to expressions displayed by individuals in other races/cultures). This effect might be due to culture-specific expression styles [25].

Cultural differences in the perception and expression of facial emotions have received much research attention (e.g., [19]), but studies on cultural differences in gaze behaviour are scarce. There is some evidence to suggest cultural variability with regard to gaze behaviour. For instance, the total amount of eye contact and the length that an individual maintains eye contact seems to vary across cultures. In Western cultures, eye contact during social interaction is considered more important than in East Asian cultures. In a study investigating the importance of different rules within social relationships, results indicated that among respondents from the U.K. and Italy, the rule ‘Should look the other person in the eye during a conversation’, was rated more important when compared to respondents from Japan and Hong Kong [26]. These rules are also reflected in overt behaviour. It has been observed that Japanese managers make less eye contact than U.S. managers during business negotiations [27], and Japanese individuals maintain less eye contact than do Canadians and Trinidadians when thinking of answers to questions [28], [29]. These results may be partly explained by the fact that within the Japanese culture, avoidance of eye contact is a sign of respect or deference [30]. Interestingly, a recent eye-tracking study that had participants view animated faces in a laboratory revealed that British participants fixated more on the mouth, whereas Japanese participants fixated mainly on the eyes [31]. Recently, an fMRI study investigated the effect of culture on the neural processing of facial images displaying fear expressions [32]. Both Japanese and U.S. White participants showed enhanced activation of the amygdala to other-culture fearful faces displaying direct vs. averted gaze. However, cultural differences emerged in amygdala responses to own-culture fearful faces. For U.S. White participants, amygdala activity was greater for averted vs. direct gaze same-culture fearful faces, whereas Japanese participants showed no significant differences in amygdala activation when viewing direct vs. averted same-culture fearful faces. The authors suggested that the lack of differential activity to own-culture direct vs. averted gaze fearful faces could be related to the cultural meaning applied to direct gaze in the Japanese culture: direct gaze could have been interpreted as a threatening cue when embedded in a fearful expression.

As results from Adams et al. [32] suggest that amygdala responses to own-culture faces displaying a direct vs. averted gaze differ between Western and Asian participants, and as the amygdala is implicated in modulating the influence of affectively salient stimuli on attention [33], an interesting question arises: are there differences in the allocation of attention resources to eye contact between individuals from Western European/North American vs. East Asian cultures? As cited above, there are cultural differences between Western European/North American and East Asian individuals in their reactions to eye contact [26]–[29]. However, it is unknown whether these attentional effects reflect voluntary attentional control processes or more automatic modulatory signals emanating from emotion-related circuits, such as the amygdala. One way to investigate this issue is to measure amygdala-mediated autonomic responses that have been associated with the orienting of attention. Orienting of attention to external stimuli is accompanied by a rapid deceleration of heart rate (HR; [34]). Lang, Bradley, and others have shown that the HR deceleration response is amplified by affectively and motivationally salient stimuli, such as unpleasant scenes or angry faces [35]–[38]. As noted above, faces displaying a direct gaze have been shown to capture and hold our visual attention [7]–[9], [39]–[40]. Thus, we should expect to find pronounced heart rate deceleration when seeing a face displaying a direct gaze.

In the present study, an autonomic correlate (i.e., heart rate deceleration) of the orienting of attention to eye contact, as well as looking time and evaluative ratings among participants originating from Western European (Finland) and East Asian (Japan) cultures, was investigated by measuring responses to direct gaze, averted gaze, and closed eyes. In the present study, ‘live’ human faces were presented as stimuli rather than pictures of faces. Our previous studies have shown that gaze direction can exert strong effects on skin conductance [10], [41], [42], as well as on electrophysiological brain responses [41], [43], when using ‘live’ faces as stimuli. In the present study, as well as in our previous studies, a computer-controlled liquid crystal window was placed between the participant and a model person for stimulus presentation. Finnish and Japanese participants faced a model person from their own cultural background. In addition to the physiological measurements, participants' looking times in different gaze direction conditions were also measured when participants were allowed to control presentation of the model face. Finally, participants were also asked to evaluate the valence and arousal of their own feelings while looking at the faces, as well as rate the stimulus faces for basic emotions, dominance, approachability, and pleasantness.

We predicted that seeing another person displaying a direct gaze would enhance HR deceleration as compared to when seeing a face with an averted gaze or closed eyes. However, the hypotheses based on culture were not that straightforward. If the differences in overt gaze behaviour between Western and Asian participants emanate from differences in voluntary control and regulation of one's attention, then it is possible that there will be no differences in HR deceleration responses to eye contact between the Western European and East Asian participants. On the other hand, if there are such differences in culture-related learning history between Western European and East Asian individuals that lead to differences at the level of (automatic) affective-motivational processing of eye contact, then differences in HR deceleration might appear. Based on available evidence, we would expect to observe more pronounced HR deceleration to eye contact in East Asian as compared to Western European participants. Less spontaneous eye contact among Japanese individuals [27]–[29] would suggest that these individuals feel that a face that is making eye contact is more unpleasant and unapproachable, but more dominant, than individuals from a Western European culture. Likewise, we would predict that Japanese participants would rate a face displaying a direct gaze as more negative than would Finnish participants. Finally, Japanese participants likely will exhibit shorter self-controlled looking-times to faces with a direct gaze as compared to Finnish participants.

## Materials and Methods

### Ethics statement

Written informed consent was obtained from all the participants. This study was approved by the Tampere Region Ethical Committee for Human Research and the Research Ethics Committee of the University of Tokyo.

### Participants

Twenty Finnish adults (10 females) and 20 Japanese adults (10 females) participated in this study (see Table 1). Both Finnish and Japanese participants were university students recruited from the University of Tampere in Finland and the University of Tokyo and other universities in Japan, respectively. All Finnish and Japanese participants were nationals of their respective countries, and no one had stayed abroad for more than six months. There were no significant group differences in chronological age (*t* = 0.63, *p* = .535). All participants completed the Social Phobia Scale (SPS; [44]) and Fear of Negative Evaluation Scale (FNE; [45]) to measure social anxiety. For Japanese participants, the Japanese versions of the SPS [46] and FNE [47] were used. For Finnish participants, these scales were translated into Finnish. There were no significant differences in scores on the SPS (*t* = 1.19, *p* = .243) or the FNE (*t* = 0.52, *p* = .608) between the Finnish and Japanese participants (Table 1). All participants had normal or corrected-to-normal visual acuity.

**Table 1:** Participants' chronological age and scores on social anxiety scales.

	Finnish (n = 20)		Japanese (n = 20)	
	M (SD)	range	M (SD)	range
**Age, years**	21.8 (3.0)	19–32	21.2 (3.5)	18–33
**SPS**	26.0 (13.1)	7–53	21.3 (12.0)	7–51
**FNE**	15.5 (6.9)	6–27	16.7 (7.7)	2–30

Means, standard deviations (SD), and range of chronological age, scores on the Social Phobia Scale (SPS) and Fear of Negative Evaluation Scale (FNE) of participants.

### Stimuli

The stimuli were faces of two Finnish females (L.P. and M.H.) and two Japanese females (S.U. and M.F.). The identity of the stimulus faces was approximately counterbalanced across the male and female participants (L.P.: six males, 4 females; M.H.: four males, 6 females; S.U.: five males, 6 females; M.F.: five males, 4 females). The faces bore a neutral expression. However, to avoid a sullen, negative face, the models maintained a very slight muscle tonus in the lower portion of their faces. The models kept their faces as motionless as possible. They tried to avoid eye blinks, but when necessary, eye blinks were allowed to occur. The faces were presented through a 30×40 cm custom-built electronic shutter with a voltage sensitive liquid crystal (LC) window (NSG UMU Products Co., Ltd.) switching from an opaque to transparent state within less than 1 ms. The shutter was attached to a frame on a table between the model and the participant. The participant was seated at a distance of 70 cm away from the frame, and the model's face was 30 cm away on the other side of the frame at the same height as the participant's face. Stimulus presentation was controlled by Neuroscan Stim software (NeuroScan, El Paso, Texas, USA) in Finland and E-Prime software (Psychology Software Tools, Inc., Pittsburgh, PA) in Japan while running on a desktop computer.

One of the authors (J.K.H.) made sure that all the conditions and procedures for handling the participants and collecting the data were as similar as possible in both laboratories (Tampere and Tokyo).

### Procedure

Upon arrival, each participant was introduced to the laboratory, the general procedure was described, and informed consent was obtained. In addition, the person who was to be the model was introduced to the participant, but no further interaction was allowed between the participant and the model at this stage. Next, two experimenters prepared the participant for the heart rate measurements. None of the participants were acquainted with the model before the experimental session to ensure that the relationship between the model and the participant would be as similar as possible for all participants. After initial preparation, the model came into the laboratory room and sat down on her side of the electronic shutter. The shutter was opened a few times during which the model adjusted her chair so that her and the participant's eyes were at the same level. The model also confirmed that the participant was seeing her adequately. During this short procedure, the model and the participant exchanged a few words.

The experiment was divided into three blocks namely, computer-controlled stimulus presentation block, self-controlled stimulus presentation block, and self-evaluative rating block. During the first block (computer-controlled presentation), the participants saw 24 trials, where the model's gaze was either direct, averted (30° left or right), or the eyes were closed (eight trials in each condition). Averted gaze was accomplished by looking at certain points on the partition behind the participant. The order of the trials was pseudo-randomised so that there were no more than two consecutive trials of the same type. Presentation time was 5000 ms. The inter-stimulus-interval (ISI) varied randomly from 17.5 to 22.5 s. During the ISI, the shutter remained opaque. A short (500 ms) and soft audio signal was presented through an earplug 5 s before the start of the next trial for the model to prepare her face and gaze direction. The model could prepare for the next gaze condition by following a printed script. The participant was instructed to look at the model.

During the second block (self-controlled presentation), only direct and averted gaze stimuli were presented (because if the model had kept her eyes closed during this block, she would not have known when the participant closed the shutter, and it would be difficult for her to prepare for the next trial). Twenty trials were presented in pseudo-randomised order, 10 trials in both conditions. The model and the stimulus conditions were otherwise the same as in the first block. The participant was instructed as follows: ‘The time that people feel it natural to look at another person's face in different situations varies. Now, we want to measure the amount of time you feel it is natural to look at the face in the present situation. There are no right or wrong answers. This is not a contest of who can stare the longest at the other person; the looking time can also be quite short’. The participant was instructed to open the shutter after hearing the soft audio signal through the speaker and close the shutter whenever he/she felt it was natural. The duration from the closing of the shutter to the time when the participants could open the shutter varied randomly from 17.5 to 22.5 s, and the soft audio signal was presented 1 s before the time when the participants could open the shutter. The model also used the soft audio signal to prepare for the next gaze condition. The participant opened and closed the shutter with a button press on a mouse, which was held in the participant's lap. The duration from the opening to the closing of the shutter was recorded with the same system used to record HR.

During the third block (self-evaluative ratings), the participants were shown one direct, one averted, and one closed eyes trial in pseudo-randomised order. On each trial, the presentation time was 5 s. Before the model opened the shutter, she said ‘Ready?’ to ensure that the participant was looking at the window when the shutter was opened. Participants were asked to rate the valence and arousal of their own feelings by using a 9-point Likert scale (valence: 1 =  pleasant, 9 =  unpleasant; arousal: 1 =  calm, 9 =  arousing), the strength of expression on the model's face along six basic emotion categories (anger, disgust, fear, happiness, sadness, and surprise) by using a 7-point Likert scale, and pleasantness, dominance, and approachability of the model's face by using a 7-point Likert scale (pleasantness: 1 =  very unpleasant, 7 =  very pleasant; dominance: 1 =  very submissive, 7 =  very dominant; approachability: 1 =  very unapproachable, 7 =  very approachable) in each condition. Each participant wrote his or her ratings down on a sheet of paper. After participants completed all the scales for one gaze direction, participants were asked to say ‘Ready’ to the model to indicate that participants were ready to be presented with the next face.

Finally, the electrodes were removed, and the participant was asked to complete the SPS and FNE questionnaires.

### HR recordings and analysis

An electrocardiogram (ECG) was recorded throughout the presentation of the stimuli with two electrodes placed on the participant's forearms. The ECG was bandpass filtered from 0.05 to 30 Hz, and the sampling rate was 500 Hz (Neuroscan/Synamps). Off-line, the data were analysed by using an in-house (Matlab-based) algorithm to identify QRS complexes (a combination of three out of five typical ECG deflections, arbitrarily named P, Q, R, S, and T-waves) in the ECG signal and to measure the time intervals between two successive R-waves (i.e. inter-beat intervals or IBI). Lengthening of the IBI corresponds to HR deceleration, and the shortening of the IBI corresponds to HR acceleration. After computer-based detection of R-peaks, the data were manually corrected for falsely detected and missing peaks. For a period between 500 ms pre-stimulus and 5000 ms post-stimulus within each trial, the IBIs were quantified and assigned to 500-ms intervals by weighting each IBI by the proportion of the 500-ms interval occupied by that IBI (see [48]). Finally, IBIs were converted to beats per minute (BPM) and averaged across different trials within each condition included in the HR analyses. Inspection of the data revealed that, in some conditions, HR slightly accelerated during the first post-stimulus 500-ms time interval in comparison to the pre-stimulus 500-ms time interval, after which HR started to decelerate in all conditions. Therefore, to be more sensitive to HR deceleration, the analyses were performed on HR change scores that were calculated by subtracting the BPMs of each post-stimulus 500-ms interval (>500 ms) from the BPM during the first post-stimulus 500-ms period. Accordingly, negative change score values indicate HR deceleration and positive values indicate HR acceleration during stimulus viewing. To reduce the number of time intervals for ANOVA analysis, HR change scores in the computer-controlled stimulus presentation block were averaged for every 1500-ms interval: between 500–2000 ms (Time 1), 2000–3500 ms (Time 2), and 3500–5000 ms (Time 3). In the self-controlled stimulus presentation block, the stimulus duration varied across participants, and 10 participants closed the shutter before 3 s in either or both conditions. Thus, HR was analysed only for 500–1000, 1000–1500, and 1500–2000 ms after the stimulus onset. In the self-controlled block, the HR data from one Finnish participant was excluded from the analysis because the participant's average looking time was less than 2 s in both conditions.

### Data analysis

To control for the effect of physical differences between the faces of the Finnish and Japanese models for each participant, we calculated a mean difference score for the HR results (in the computer-controlled stimulus presentation block) and rating scores by subtracting the mean value for the closed eyes condition from those for the direct or averted gaze conditions. This made it possible to investigate the effects of gaze direction without any relation to differences in the models' facial appearance and expressions. This was important for analysing cultural differences. Because there was no closed eyes condition in the self-controlled stimulus presentation block, the HR and looking times in this block were simply averaged in both conditions (direct and averted) for each participant. The mean looking times and rating scores were analysed by using a two-way ANOVA with culture (FI, Finnish or JP, Japanese) as the between-participant factor and gaze (direct or averted) as the within-participant factor. The HR in the computer-controlled and self-controlled stimulus presentation blocks were analysed by using a three-way ANOVA with culture as a between-participant factor and gaze and time (Time 1: 500–2000 ms, Time 2: 2000–3500 ms, or Time 3: 3500–5000 ms in the computer-controlled block; 500–1000 ms, 1000–1500 ms, or 1500–2000 ms in the self-controlled block) as within-participant factors. Bonferroni-adjusted *p*-values were used to control for multiple comparisons.

## Results

### Heart rate

#### Computer-controlled stimulus presentation block

**Figure 1:**
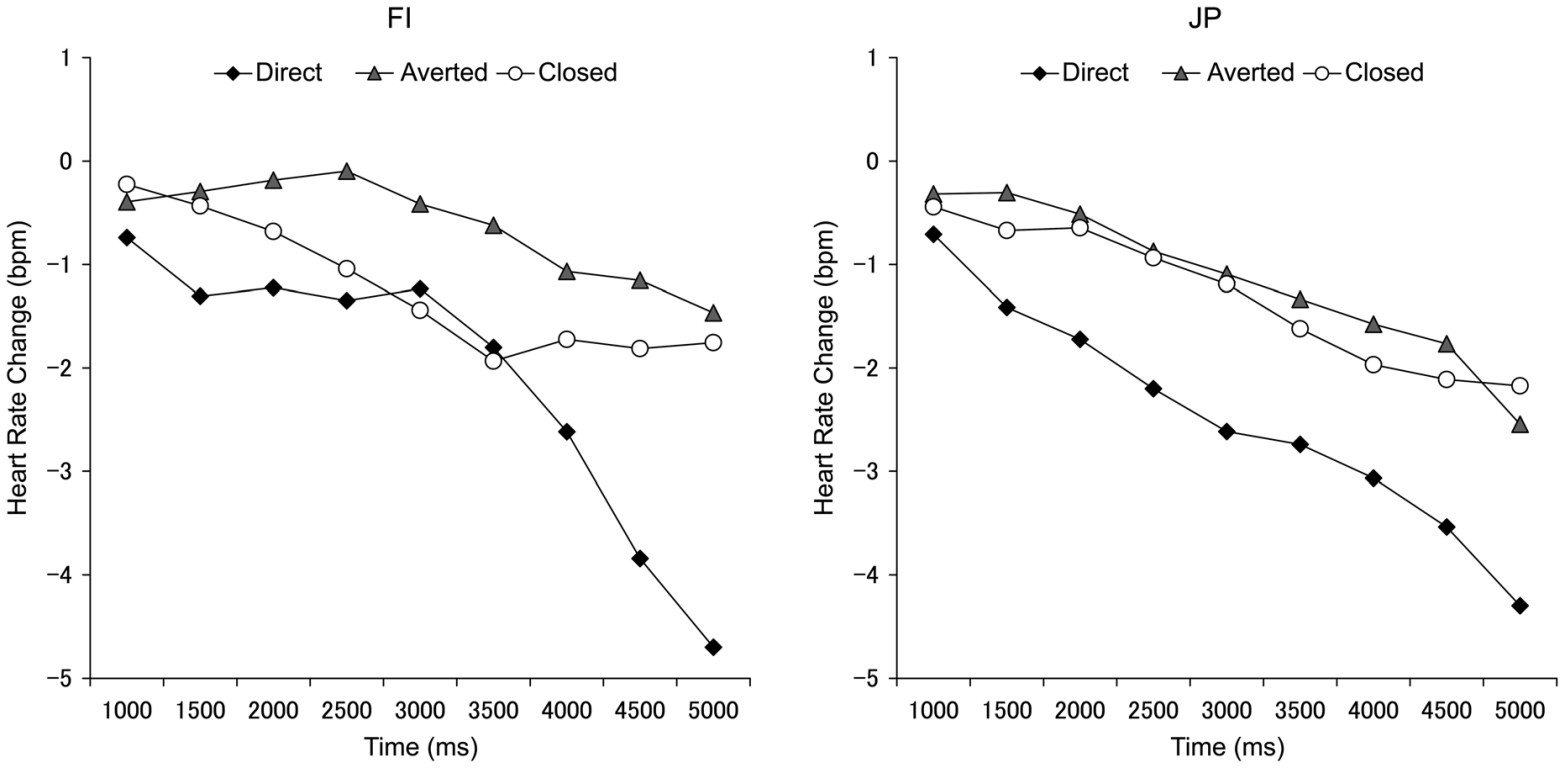
Heart rate change in the computer-controlled stimulus presentation block. The mean heart rate change of direct gaze (black square dots), averted gaze (grey triangle dots), and closed eyes condition (white circular dots) in the computer-controlled stimulus presentation block in the Finnish (left) and Japanese groups (right). The following abbreviations are used: FI, Finnish; JP, Japanese.

**Figure 2:**
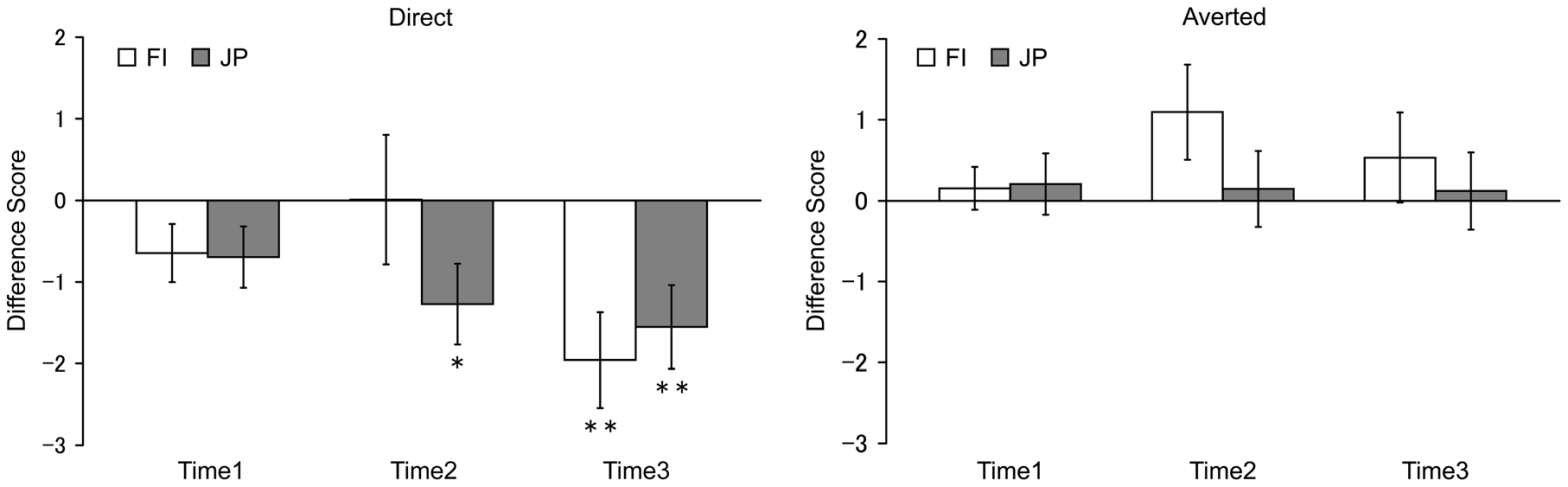
Heart rate difference scores in the computer-controlled stimulus presentation block. The mean heart rate difference scores of direct gaze (left) and averted gaze (right) in the computer-controlled stimulus presentation block in the FI group (white bars) and the JP group (grey bars). The following abbreviations are used: FI, Finnish; JP, Japanese; **p<.01, *p<.05; Asterisks below error bars indicate significant difference from zero. Error bars represent standard errors.

Our results showed that HR decelerated after the presentation of a face displaying a direct gaze (see Figure 1). As described in Materials and Methods, statistical analyses were performed on difference scores within three time-windows (Figure 2). The ANOVA analysis showed a significant main effect of gaze (*F* (1, 38)  = 16.51, *p*<.001, *η_p_^2^* = .30); HR deceleration was significantly greater when viewing a direct gaze (*M* = −1.02, *SEM*  = 0.30) than when viewing an averted gaze (*M* = 0.38, *SEM*  = 0.28). There was also a significant interaction between gaze and time (*F* (2, 76)  = 6.03, *p* = .004, *η_p_^2^* = .14). A simple effects analysis revealed a significant simple main effect of time for the direct gaze difference scores (*F* (2, 37)  = 9.88, *p*<.001, *η_p_^2^* = .35); HR deceleration was greater at Time 3 (*M* = −1.75, *SEM*  = 0.38) compared to Time 1 (*M* = −0.67, *SEM*  = 0.25) and Time 2 (*M* = −0.63, *SEM*  = 0.47) (both *p*s <.05). The simple main effect of time was not significant for the averted gaze difference scores (*p*>.05). Other main effects and interactions were not significant (all *p*s >.05).

Additionally, we assessed *t*-tests to determine whether the HR difference scores deviated from zero for both the direct and averted gaze conditions within each culture. For the averted gaze condition, difference scores did not differ from zero in either culture. In other words, HR decelerated similarly for the averted gaze and closed eyes conditions. For the direct gaze condition, HR of the JP participants significantly decreased at Time 2 (*t* = 2.64, *p* = .016, *d* = 1.21; *M* = −1.27, *SEM*  = 0.50) and Time 3 (*t* = 3.10, *p* = .006, *d* = 1.42; *M* = −1.55, *SEM*  = 0.51) in comparison to the closed eyes condition. For FI participants, HR during the direct gaze condition significantly decreased only at Time 3 (*t* = 3.34 *p* = .003, *d* = 1.53; *M* = −1.96, *SEM*  = 0.59). This suggests that HR deceleration was faster for the JP than the FI participants when looking at a face displaying a direct gaze. However, this result should be interpreted with caution because there was no significant interaction between culture, gaze, and time (*F* (2, 76)  = 1.43, *p* = .246) in the 3-way ANOVA.

#### Self-controlled stimulus presentation block

**Figure 3:**
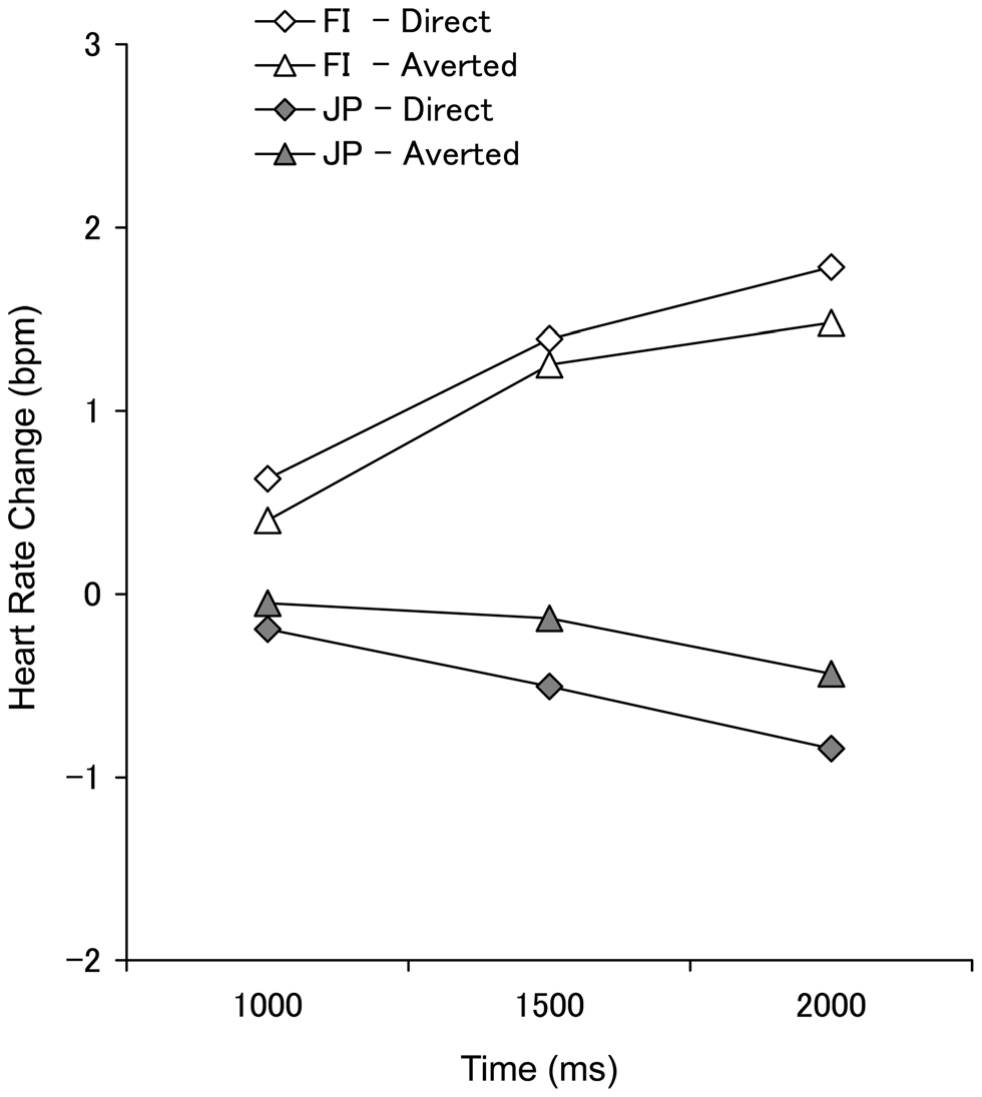
Heart rate change in the self-controlled stimulus presentation block. The mean heart rate change for direct (square dots) and averted gaze (triangle dots) in the self-controlled stimulus presentation block in the FI (white dots) and the JP group (grey dots). The following abbreviations are used: JP, Japanese; FI, Finnish.

For the JP participants, HR decelerated in the self-controlled stimulus presentation block (*M* = −0.36, *SEM*  = 0.39) as was the case during the computer-controlled block; however, the FI participants showed an accelerated HR (*M* = 1.16, *SEM*  = 0.33) during this block (Figure 3). The main effect of culture was significant (*F* (1, 37)  = 8.74, *p* = .005, *η_p_^2^* = .19). There was also a significant interaction between culture and time (*F* (2, 74)  = 7.47, *p* = .001, *η_p_^2^* = .17). A simple effects analysis revealed a significant main effect of time in the FI group (*F* (2, 36)  = 6.98, *p* = .003, *η_p_^2^* = .28) but not in the JP group (*F* (2, 36)  = 1.05, *p* = .359, *η_p_^2^* = .06); HR in the 1000–1500 ms (*M* = 1.32, *SEM*  = 0.34) and 1500–2000 ms (*M* = 1.63, *SEM*  = 0.51) intervals was increased compared to the 500–1000 ms (*M* = 0.51, *SEM*  = 0.19) interval within the FI group (both *p*s <.05). Other main effects and interactions were not significant (all *p*s >.05).

### Looking times

An ANOVA revealed a significant main effect of gaze (*F* (1, 38)  = 7.02, *p* = .012, *η_p_^2^* = .16); participants in both groups looked at faces for a shorter duration in the direct gaze condition (*M* = 5.82s, *SEM*  = 0.58) as compared to the averted gaze condition (*M* = 6.74s, *SEM*  = 0.77). The main effect of culture and the interaction between gaze and culture were not significant (both *p*s >.05).

### Ratings

#### Subjective valence and arousal

For valence difference scores (direct/averted gaze – closed eyes), there was no significant effect of gaze, culture, or an interaction between the two (all *p*s >.05). For arousal difference scores, an ANOVA revealed a significant main effect of gaze (*F* (1, 38)  = 21.75, *p*<.001, *η_p_^2^* = .36), indicating that faces displaying a direct gaze (*M* = 2.50, *SEM*  = 0.34) were rated as more arousing than faces displaying an averted gaze (*M* = 0.80, *SEM*  = 0.22). The main effect of culture and the interaction between culture and gaze were not significant (both *p*s >.05).

#### Models' facial expressions

**Figure 4:**
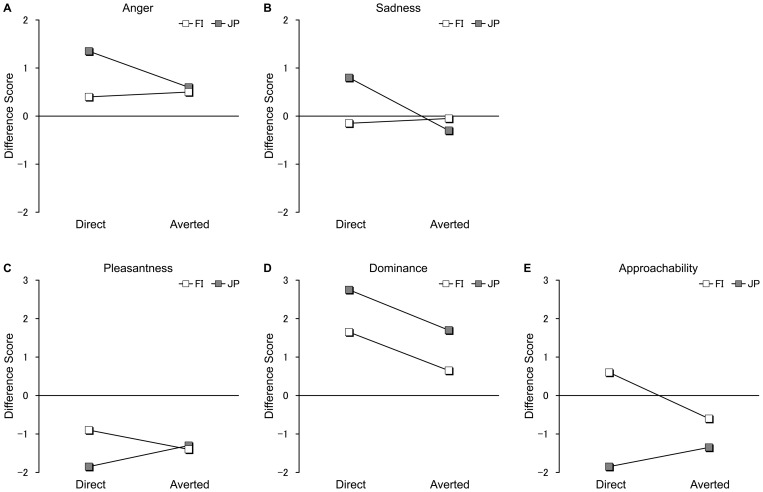
Evaluative rating difference scores. The mean difference scores of direct and averted gaze for anger (A), sadness (B), pleasantness (C), dominance (D), and approachability rating scores (E) in the FI (white dots) and JP groups (grey dots). The following abbreviations are used: FI, Finnish; JP, Japanese.

For anger, there was a significant main effect of culture (*F* (1, 38)  = 5.19, *p* = .028, *η_p_^2^* = .12); JP participants (*M* = 0.98, *SEM*  = 0.18) rated the direct and averted gaze faces (with respect to closed eyes faces) as angrier than did FI participants (*M* = 0.45, *SEM*  = 0.14) (Figure 4A). Importantly, there was also a significant interaction between culture and gaze (*F* (1, 38)  = 4.19, *p* = .048, *η_p_^2^* = .10). A simple effects analysis revealed a significant simple main effect of gaze in the JP (*F* (1, 38)  = 6.52, *p* = .015, *η_p_^2^* = .15) but not in the FI group (*F* (1, 38)  = 0.12, *p* = .735, *η_p_^2^*<.01). In the JP group, direct gaze faces (*M* = 1.35, *SEM*  = 0.26) were rated as more angry than faces with an averted gaze (*M* = 0.60, *SEM*  = 0.24). Additionally, there was a significant simple main effect of culture for the direct gaze (*F* (1, 38)  = 9.70, *p* = .003, *η_p_^2^* = .20) but not for the averted gaze difference scores (*F* (1, 38)  = 0.10, *p* = .753, *η_p_^2^*<.01). JP participants (*M* = 1.35, *SEM*  = 0.26) rated faces displaying a direct gaze as more angry than did FI participants (*M* = 0.40, *SEM*  = 0.15). The main effect of gaze was not significant (*p* = .126).

For sadness evaluations (Figure 4B), an interaction between culture and gaze was observed (*F* (1, 38)  = 5.49, *p* = .024, *η_p_^2^* = .13), and there was a simple main effect of gaze in the JP group (*F* (1, 38)  = 9.23, *p* = .004, *η_p_^2^* = .20) but not in the FI group (*F* (1, 38)  = 0.08, *p* = .784, *η_p_^2^*<.01). JP participants rated direct gaze faces as sadder (*M* = 0.80, *SEM*  = 0.43) than averted gaze faces (*M* = −0.30, *SEM*  = 0.38). The main effects of culture and gaze were not significant (both *p*s >.05).

For evaluations of disgust, fear, happiness, and surprise, there were no significant effects of gaze and culture and no significant interaction between the two (all *p*s >.05).

#### Model faces' pleasantness, dominance, and approachability

For pleasantness (Figure 4C), an ANOVA revealed a significant interaction between culture and gaze (*F* (1, 38)  = 4.37, *p* = .043, *η_p_^2^* = .10). A simple effects analysis revealed a marginal simple main effect of culture for the direct gaze difference scores (*F* (1, 38)  = 2.95, *p* = .094, *η_p_^2^* = .07), suggesting that JP participants rated faces displaying a direct gaze (*M* = −1.85, *SEM*  = 0.42) as more unpleasant than did FI participants (*M* = −0.90, *SEM*  = 0.35). The main effects of culture and gaze were not significant (both *p*s >.05).

For dominance (Figure 4D), there were significant main effects of culture (*F* (1, 38)  = 11.32, *p* = .002, *η_p_^2^* = .23) and gaze (*F* (1, 38)  = 14.39, *p* = .001, *η_p_^2^* = .27). This indicated that JP participants (*M* = 2.23, *SEM*  = 0.18) rated the models' faces as more dominant than did FI participants (*M* = 1.15, *SEM*  = 0.26), and faces displaying direct gaze (*M* = 2.20, *SEM*  = 0.25) were rated more dominant than faces displaying an averted gaze (*M* = 1.18, *SEM*  = 0.20). The interaction between culture and gaze was not significant (*p* = .927).

For approachability (Figure 4E), an ANOVA revealed a significant main effect of culture (*F* (1, 38)  = 14.78, *p*<.001, *η_p_^2^* = .28), indicating that FI participants (*M* = 0.00, *SEM*  = 0.32) rated the models' faces as more approachable than did JP participants (*M* = −1.60, *SEM*  = 0.27). There was also a significant interaction between culture and gaze (*F* (1, 38)  = 12.18, *p* = .001, *η_p_^2^* = .24). A simple effects analysis revealed a simple effect of culture for the direct gaze difference scores (*F* (1, 38)  = 20.49, *p*<.001, *η_p_^2^* = .35), as well as a simple effect of gaze for the FI group (*F* (1, 38)  = 12.13, *p* = .001, *η_p_^2^* = .24). FI participants (*M* = 0.60, *SEM*  = 0.38) rated the direct gaze face as more approachable than did JP participants (*M* = −1.85, *SEM*  = 0.39), and FI participants also rated the direct gaze face as more approachable than the averted gaze face (*M* = −0.60, *SEM*  = 0.29). The main effect of gaze was not significant (*p* = .159).

## Discussion

To our knowledge, the present study is the first to investigate cultural differences between East Asian (Japanese) and Western European (Finnish) participants in the processing of another, own-culture person's live face with direct (eye contact) and averted gaze by measuring a combination of physiological (HR; heart rate), behavioural (looking times), and subjective experiential (ratings regarding participant's own feelings as well as impressions of the model's face) responses. Specifically, the focus of the present study was on potential cultural differences in the eye contact effect that might emerge as an interaction between culture and gaze direction. However, cultural differences in response to different gaze directions were not observed within physiological or behavioural responses. In a condition where the presentation of the facial stimuli was controlled by a computer (for 5-s periods), faces displaying a direct gaze elicited more pronounced HR deceleration than faces displaying an averted gaze within both cultural groups. Instead, in a condition where participants were able to control the stimulus presentation and duration, both direct and averted gaze faces elicited decelerated HR among Japanese participants but accelerated HR among Finnish participants. In this condition, gaze direction had no effect on HR in either cultural group. Moreover, both Finnish and Japanese participants spent less time looking at a direct gaze face than an averted gaze face. Cultural differences regarding the effects of gaze direction were observed in the self-evaluative ratings. Japanese individuals rated the direct gaze face as more unapproachable and unpleasant than did Finnish individuals. Regarding the facial expression ratings (which bore a neutral expression), Japanese individuals rated the direct gaze faces as angrier than did Finnish individuals, and Japanese participants also rated direct gaze faces as angrier and sadder than when viewing averted gaze faces. There were no cultural differences in facial expression ratings using other emotion categories, suggesting that Japanese participants did not simply feel that eye contact would result in enhanced impressions of all negative emotions. In both cultures, gaze direction influenced participants' subjective ratings of arousal, as well as the model faces' dominance; faces displaying a direct gaze were rated as more arousing and dominant than faces displaying an averted gaze. In addition, there were cultural differences in dominance ratings irrespective of gaze direction; Japanese participants rated direct gaze faces and averted gaze faces as more dominant than did Finnish participants.

The use of the electronic shutter enabled us to measure HR to live faces in a well-controlled condition. In the computer-controlled stimulus presentation block, results revealed a strong effect of gaze direction, which was unaffected by participants' cultural background. In both cultures, faces displaying a direct gaze elicited more pronounced HR deceleration than faces displaying an averted gaze. These results suggest that the cultural differences in eye contact behaviour reported in previous studies (e.g., [28], [29]) might be driven by differences in strategic control and attentional regulation rather than differences in reflexive attention to other persons' gaze that is adapted to the cultural environment. HR deceleration is thought to indicate a sensory intake or orienting response to a stimulus ([34]; see [35] for a review). For example, prominent HR deceleration has been reported as a response to emotionally negative pictures, such as an aimed gun [36], snakes, and spiders [49], all of which usually elicit attentional orienting. HR deceleration has also been shown when participants view facial stimuli. Socially threatening faces (i.e., angry faces) have been shown to elicit decelerated HR [37], [50], and Peltola and colleagues [51] showed that 7-month-old infants exhibited HR deceleration specifically to fearful faces, indicating that facial expressions of fear start to orient humans' attention at an early age. Based on this attentional orienting account of HR deceleration, the present HR results during the computer-controlled stimulus presentation block could be interpreted as eye contact eliciting strong orienting responses across cultures. This finding extends previous research which showed that eye contact captures visual attention [7], [8]; our results suggest that the enhanced attentional orienting to eye contact can be observed at the physiological level, and this orientation is culturally independent.

It should be noted that there are previous studies investigating the effects of gaze direction on HR and, in fact, a typical finding in those studies has been that HR accelerates in response to eye contact [11], [52]. In these studies, another individual continuously observed participants in a direct gaze condition (i.e., 4 min or 50 prisoner's dilemma trials), and results showed an averaged HR throughout the condition. However, HR usually decelerates first after the emotional stimulus onset and accelerates thereafter (e.g., [37], [49]). Thus, these previous studies [11], [52] did not investigate HR change immediately after eye contact onset and, therefore, failed to observe HR deceleration in response to eye contact. Interestingly, a recent study showing animated faces with direct and averted gaze reported that the male but not the female participants showed larger HR deceleration to direct vs. averted gaze during a 4–s time-window [53]. Wieser and colleagues [54] also investigated HR in response to gaze direction of animated faces, and the authors analysed HR data within two different time-windows. In a time-window between 3500 and 6500 ms post-stimulus onset, HR accelerated as compared to a baseline level and for individuals who scored high on a measure of social anxiety HR acceleration was also greater when viewing a face with a direct rather than an averted gaze. In a time-window between 500–3500 ms post-stimulus onset, results showed HR deceleration (similar to the present study), but in Wieser et al.'s study, gaze direction failed to exert a statistically significant effect on this HR deceleration response. One possible reason for the discrepancies between the present and the previous studies may be related to differences in the stimuli used. Both in Wieser et al.'s study [54] and in Soussignan et al.'s study [53] animated faces were used as stimuli, whereas faces of another person were used in the present study. In our previous studies, live faces displaying a direct gaze elicited larger SCRs, greater relative left-sided frontal EEG asymmetry [41], [42], and larger face-sensitive N170 amplitudes [43] than faces displaying an averted gaze. Conversely, gaze direction within pictures of the same faces presented on a computer monitor did not have an effect on these measures. Given these results, the animated eye contact faces, such as those used in the previous studies [53], [54] might not be socially powerful enough to exert an influence on attentional orienting and corresponding autonomic responses.

There was no effect of gaze direction on HR in the self-controlled stimulus presentation block. Interestingly, there was a main effect of participants' cultural background on HR responses. HR of Japanese participants decelerated as it did in the computer-controlled stimulus presentation block, but there was a HR acceleration response among Finnish participants. These results might reflect cultural differences in the way participants reacted to the ‘self-control’ (i.e., controllability). Japanese individuals, even when they could control the stimulus presentation, might have exhibited a strong orienting reflex to the appearance of another person's face. Socially threatening faces (i.e., angry faces) have been shown to elicit decelerated HR and reduced body sway, interpreted as a defensive and freezing response [50]. By contrast, the Finnish participants might not have shown an orienting reflex to the appearance of the face when they could control the stimulus presentation. Instead, Finnish participants might have shown HR acceleration that was mediated by sympathetic arousal. This finding is compatible with a previous study assessing Finnish participants where a similar electronic shutter, and similar computer-controlled vs. self-controlled stimulus presentation blocks, was applied while measuring sympathetic activity with skin conductance responses [10]. In that study, although larger SCRs were elicited by direct vs. averted gaze faces in the self-controlled stimulus presentation condition, SCRs were larger, overall, in the self-controlled vs. computer-controlled stimulus presentation condition. We speculate that, perhaps, gaining controllability over seeing the model person may have enhanced Finnish participants' awareness of their role in the interaction with the model and, consequently, increased sympathetic arousal. This did not appear to be the case for Japanese participants. The observed pattern of results might even be linked with Japanese participants' propensity for shyness (e.g., [55], [56]), which might have interfered with the awareness of Japanese participants' interactive role with the model. However, it is difficult to draw a strong conclusion from the main effect of cultural background, as we did not have non-social control stimuli; therefore, we cannot identify the specificity or generalizability of the results to other forms of stimuli.

In both cultures, direct gaze faces were viewed for a shorter duration than averted gaze faces. This result replicates and extends results of a previous study with Finnish participants using a similar methodology [10]. Regarding earlier studies showing differences between Western European/North American and East Asian participants in their reactions to eye contact [26]–[29], it was rather surprising that cultural background did not interact with the gaze direction effect in the current study. However, the lack of this interaction should be interpreted with caution. For example, in the present study, the dependent measure was the length of time participants kept the shutter open. Because an eye-tracking device did not record participants' eye gaze fixations, the measured looking times do not necessarily correspond to the duration of actual eye contact. Additionally, viewing another person through an LC window, and controlling its opening and closing, can hardly be considered a natural interaction. Studies investigating eye fixation patterns while scanning static facial expression images ([20]; but see also [57]), and moving animated faces [31] have not observed shorter fixation durations to eye regions between samples of East Asian vs. Western European individuals. Thus, it is possible that cultural differences in the duration of spontaneous eye contact might be observed in more natural situations (e.g., [27]–[29]) but not in studies that present faces as discrete visual stimuli, whether those stimuli are static or dynamic images (e.g., [20], [31]) or real faces (the present study). Further studies that test this hypothesis directly will be beneficial.

The gaze direction of the models differentially affected various subjective ratings between Japanese and Finnish participants, which might relate to different cultural norms regarding the use of eye contact. Japanese participants rated the model as angrier when the model displayed a direct rather than an averted gaze, whereas gaze direction had no effect on Finnish participants' anger ratings. Instead, Finnish participants rated direct eye contact as more approachable than displays of averted gaze; however, gaze direction had no effect on Japanese participants' approachability ratings. In addition to anger, Japanese individuals also rated the model as sadder when displaying a direct gaze as compared to an averted gaze. Among Finnish participants, gaze direction had no effect on sadness ratings. These results suggest that gaze direction might be part of the emotional display of anger and sadness in the Japanese culture, and this, in turn, possibly affected the subjective evaluations of approachability and pleasantness. By contrast, the present results suggest that both Japanese and Finnish participants rated direct gaze faces as more arousing than averted gaze faces. This result replicates findings from previous studies using the same methodology with Finnish samples [41], [43]. Additionally, participants from both cultures rated the direct gaze faces as more dominant than the averted gaze faces. Finally, we did not observe any effects of culture, gaze direction, or the interaction between the two on subjective valence ratings. Taken together, the present rating results imply that cultural differences in spontaneous eye contact behaviour (e.g., [27]–[29]) may result from differences in cultural norms regarding eye contact behaviour (e.g., [26]) rather than from differences in autonomic reactions and subjective arousal in response to eye contact.

In addition to cultural differences, ethnic differences might have influenced the present results among the Japanese and Finnish samples. To solve this issue, future studies are needed to compare individuals of the same ethnic origin but raised in different cultural backgrounds, as well as comparing individuals coming from the same culture but who differ with respect to their ethnic background. In addition, it has been suggested that there are culture-specific facial emotion recognition and expression styles [19], [25]. Thus, although the effect of physical differences between the models' faces in both cultures was controlled in the present study by using difference scores, studies that have participants view both own- and other-group faces will be necessary. It should also be noted that, in the present study, we collected data from individuals representing only one Western and one East Asian country. Thus, we do not know to which extent the present results generalize to Western and East Asian cultures, in general. Therefore, further studies investigating individuals coming from different geographic regions and countries within Western and East Asian cultures would be needed to extend the present result. Finally, in the self-controlled stimulus presentation block, our methodology did not provide sharp enough measurements as to where participants actually looked when the shutter was open. In the future, an eye-tracking device should be used when viewing real faces to investigate the relationship between eye contact behaviour, physiological responses, and subjective feelings to eye contact.

## Conclusions

The current study investigated cultural differences (East Asian vs. Western Europeans) in response to eye contact with another (real) person. The results revealed that eye contact elicited stronger heart rate deceleration responses, shorter looking times, and higher ratings of subjective arousal as compared to a face displaying an averted gaze in both cultures. By contrast, cultural differences related to eye contact were observed in various evaluative responses (e.g., facial emotion, approachability etc.) to the models presenting different gaze directions. These results suggest that cultural differences in eye contact behaviour mainly emerge from differential display rules and cultural norms rather than from the effects of culture on the development of behavioural and physiological responses to direct gaze. The rating results suggest that individuals from an East Asian culture perceive another's face as angrier and more unapproachable and unpleasant when making eye contact as compared to individuals from a Western European culture. The rating results also suggest that direct vs. averted gaze can have differential influences on perceptions about another person's facial affect and disposition. Given that eye contact is crucial for daily interpersonal communication, the current results provide unique insight into how we behave in front of others. These results also have the potential for facilitating effective cross-cultural communication. For example, East Asian individuals should not overinterpret the eye contact of Western European individuals as signalling anger, and Western European individuals should tolerate shorter and less frequent eye contact with East Asian individuals, as East Asian individuals might think that long and frequent eye contact could present an unapproachable impression. Further studies are necessary to better understand how cultural norms modulate eye contact behaviours.
